# Initial combination anti-viral therapy with lamivudine and adefovir dipivoxil decreases short-term fatality rate of hepatitis-B-virus-related acute-on-chronic liver failure

**DOI:** 10.1186/s12985-015-0323-3

**Published:** 2015-06-24

**Authors:** Jiahong Yang, Gao Chen, Xuebing Chen, Hao Zhang, Di Jiang, Guang Yang

**Affiliations:** Department of Infectious Diseases, People’s Hospital of Deyang City, No. 173, Taishan North Road, Deyang, 618000 Sichuan Province China

**Keywords:** Hepatitis B virus, Acute-on-chronic liver failure, Antiviral treatment, Lamivudine, Adefovir dipivoxil

## Abstract

**Background:**

Acute-on-chronic liver failure (ACLF) is a common serious hepatitis B virus (HBV)-related disease and has a poor prognosis. Until recently, initial combination antiviral treatment in ACLF patients was rarely reported. This study evaluated the effect of initial combination treatment with lamivudine and adefovir dipivoxil on the prognosis of HBV-related ACLF.

**Methods:**

In this retrospective study, 131 eligible ACLF patients, including 61 treated with 100 mg lamivudine and 10 mg adefovir dipivoxil daily and 70 not treated with any nucleoside analogs (NAs), were selected and assigned into the NA and non-NA groups. All the patients received standard medicinal therapy. At weeks 0–4 and 12, serum markers for hepatic and renal functions were measured in all patients and accumulated fatality rates were calculated. Statistical analyses, including Student’s *t* test, *χ*^2^ test and unconditional logistic regression analysis, were performed using SPSS version 17.0 software.

**Results:**

Clinical data indicated that improvement of hepatic function was better in the NA than in the non-NA group. The accumulated fatality rate in the NA group was lower than in the non-NA group at weeks 2–4 and 12, and these differences were significant. Univariate analysis showed that age, prothrombin activity, model of end-stage liver disease (MELD) score, and treatment without NAs were risk factors for short-term survival of ACLF. Further research by unconditional logistic regression analysis identified that older age, high MELD score and treatment without NAs were independent risk factors for short-term survival of ACLF.

**Conclusions:**

Initial combination antiviral treatment is effective in decreasing short-term fatality of HBV-related ACLF.

## Introduction

Chronic hepatitis B virus (HBV) infection is a major public health problem worldwide. According to estimation of the World Health Organization, about 400 million people are chronically infected with HBV and ~1 million die from HBV-related diseases each year [1, 2]. Chronic HBV infection leads to several related liver diseases, including chronic hepatitis B, cirrhosis, hepatocellular carcinoma (HCC), and liver failure [3]. Acute-on-chronic liver failure (ACLF), defined in 1995 as a condition in which acute and chronic liver disease occur simultaneously, is a common serious HBV-related liver disease and causes acute deterioration of liver function [4]. ACLF has poor prognosis [5, 6].

There is no standard treatment for ACLF. The curative effect of standard medical treatment is limited and the fatality rate is high [7–9]. Clinical application of an artificial liver support system can temporarily improve patients’ condition. However, it is not effective in decreasing fatality rate [10–13]. Although liver transplantation is effective for ACLF, its clinical application is hindered by the difficulty in finding suitable donors and its high cost [14, 15]. Thus, a new treatment to improve prognosis is eagerly required in clinical practice.

Although the mechanism of HBV-related ACLF remains obscure, growing evidence suggests that viral factors [16–18], host factors [19–21], and their interactions determine the outcome of ACLF. HBV DNA replication is an important factor for deterioration of liver function and closely associated with the risk of HCC.

Nucleoside analogues (NAs) can inhibit the replication of HBV and decrease serum HBV DNA loads, leading to improvement of liver function and reduction of cirrhotic complications and occurrence of HCC [22–24]. Treatment with one NA alone is unsatisfactory, although monotherapy can improve patients’ condition and decrease short-term and long-term fatality rates [7–9]. Previous research has suggested that initial combination antiviral therapy with lamivudine and adefovir dipivoxil is more effective than monotherapy in decreasing serum HBV load and improving prognosis [25, 26]. Until recently, initial combination antiviral therapy in ACLF patients was rarely reported. ACLF develops rapidly and has poor prognosis, and combination antiviral therapy is more effective than monotherapy [25, 26]. Therefore, in the present study, 61 patients were treated with 100 mg lamivudine and 10 mg adefovir dipivoxil daily and 70 were not treated with any NA. Patients were selected to assess the effect of initial combination antiviral therapy on clinical condition and short-term survival of ACLF.

## Results

### Baseline characteristics

As mentioned above, 131 eligible ACLF patients were recruited and divided into NA and non-NA groups. There were no significant differences in the baseline characteristics of age, sex, hepatitis B e antigen (HBeAg) status, model of end-stage liver disease (MELD) score, and baseline levels of serum HBV DNA load, total bilirubin (TBIL), albumin, alanine aminotransferase (ALT), international normalized ratio (INR), prothrombin activity (PTA) and creatinine between the two groups (Table 1).

**Table 1:** Baseline characteristics of the patients

	NA group (mean ± SD)	Non-NA group (mean ± SD)	*t* or *χ* ^2^ value	*P* value
Male (%)	52 (85.25)	59 (84.29)	0.02	0.88
HBeAg positive (%)	19 (31.15)	18 (25.71)	0.48	0.49
Age (yr)	40.31 ± 10.75	43.14 ± 10.37	-1.53	0.13
HBV DNA log_10_ (copies/ml)	5.72 ± 1.38	5.31 ± 1.56	1.53	0.13
TBIL (μmol/L)	278.56 ± 116.09	260.24 ± 115.50	0.90	0.37
Albumin (g/L)	34.73 ± 5.85	34.35 ± 4.49	0.42	0.67
ALT (U/L)	1037.44 ± 823.50	833.93 ± 794.70	1.44	0.15
PTA (%)	31.15 ± 9.71	33.34 ± 10.20	-1.25	0.21
Creatinine (μmol/L)	66.01 ± 12.46	76.94 ± 52.23	-1.70	0.09
MELD Score	22.82 ± 2.99	23.95 ± 5.91	-1.40	0.17

### Serum TBIL, ALT, albumin and PTA at different times of therapy

Baseline serum TBIL, ALT, albumin and PTA did not differ significantly between the NA and non-NA groups. Serum TBIL levels increased at week 1, decreased below baseline levels at week 2, and then continued to decrease in the NA group (Table 2). In the non-NA group, serum TBIL levels continued to increase, reached a peak at week 2, and then began to decrease. Serum TBIL levels were lower in the NA group than non-NA group at weeks 1–4 and these differences were significant at weeks 2 and 3 (*t* = 4.52, *P* < 0.001; *t* = 3.59, *P* = 0.001) (Table 2). In the NA group, serum albumin levels continued to increase. In the non-NA group, serum albumin levels decreased below baseline levels at week 1 and then fluctuated at weeks 2–4. Serum albumin levels were higher in the NA group than non-NA group at weeks 1–4 and theses differences were significant (*t* = 3.67, *P* < 0.001; *t* = 3.92, *P* < 0.001; *t* = 5.77, *P* < 0.001; *t* = 3.88, *P* < 0.001) (Table 2). Although serum PTA levels continued to increase in the NA and non-NA groups, it increased more rapidly in the NA group, and serum PTA levels were higher in the NA than non-NA group at weeks 1–4. These differences were significant at weeks 2 and 3 (*t* = 2.52, *P* = 0.013; *t* = 2.38, *P* = 0.020). Serum ALT levels continued to decrease in these two groups although the differences between the groups were not significant. There was no patient with abnormal serum creatinine and urea nitrogen levels in either group until week 12.

**Table 2:** Time-dependent changes of serum TBIL, ALT, albumin and PTA

Serum marker	Time	NA group	Non-NA group	*t* value	*P* value
TBIL (μmol/L)	Week 1	310.98 ± 134.94	360.39 ± 122.11	1.95	0.054
	Week 2	272.74 ± 166.83	427.22 ± 189.74	4.52	<0.001
	Week 3	239.22 ± 172.03	379.72 ± 200.63	3.59	0.001
	Week 4	240.55 ± 210.19	299.47 ± 195.52	1.10	0.28
ALT (U/L)	Week 1	224.43 ± 234.97	267.75 ± 242.43	-0.93	0.36
	Week 2	120.20 ± 116.55	122.05 ± 89.96	-0.09	0.93
	Week 3	87.31 ± 74.70	73.96 ± 38.98	1.02	0.31
	Week 4	63.62 ± 49.75	57.48 ± 28.60	0.56	0.58
Albumin (g/L)	Week 1	35.31 ± 5.09	32.05 ± 3.74	3.67	<0.001
	Week 2	36.78 ± 5.02	33.37 ± 3.78	3.92	<0.001
	Week 3	37.51 ± 5.41	32.39 ± 2.90	5.77	<0.001
	Week 4	37.38 ± 5.21	32.42 ± 4.41	3.88	<0.001
PTA (%)	Week 1	52.77 ± 27.31	45.59 ± 24.93	1.34	0.18
	Week 2	57.85 ± 29.73	44.31 ± 23.20	2.52	0.013
	Week 3	65.58 ± 30.56	49.70 ± 27.53	2.38	0.020
	Week 4	58.23 ± 31.56	48.50 ± 24.78	1.14	0.260

### Accumulated fatality rates in the two groups

All ACLF patients in these two groups were followed up to week 12. At week 12, 55 patients were dead and the fatality rate was 41.98 %. Accumulated fatality rate at weeks 2–4 and 12 in the NA group was 1.64 % (1/61), 14.75 % (9/61), 21.31 % (13/61) and 24.59 % (15/61), respectively. In the non-NA group, accumulated fatality rate was 21.43 % (15/70), 35.71 % (25/70), 47.14 % (33/70) and 57.14 % (40/70) at weeks 2–4 and 12, respectively. The accumulated fatality rates in the NA group were lower than in the non-NA group and these differences were significant (*χ*^2^ = 11.91, *P* = 0.001; *χ*^2^ = 7.45, *P* = 0.006; *χ*^2^ = 9.55, *P* = 0.002; *χ*^2^ = 14.18, *P* < 0.001). This indicated that antiviral therapy may decrease short-term fatality rate.

### Factors influencing short-term survival of ACLF

Univariate analysis was performed to identify risk factors for short-term survival of ACLF patients. Age, PTA, MELD score and treatment without NAs were risk factors for short-term survival of ACLF patients (*t* = 3.06, *P* = 0.003; *t* = 2.05, *P* = 0.042; *t* = 3.49, *P* = 0.001; *χ*^2^ = 14.18, *P* < 0.001) (Table 3). Multivariate analysis using unconditional logistic regression was performed to identify independent risk factors for short-term survival of ACLF patients and *P* ≥ 0.05 was selected as the exclusion criterion. Sex, age, HBeAg status, HBV DNA loads, MELD score, serum TBIL level, ALT level, albumin level and PTA level, and treatment without NAs were included in the unconditional regression analysis. Unconditional logistic regression analysis showed that age, MELD score and treatment without NAs were independent risk factors for short-term survival of ACLF [*P* = 0.007, odds ratio (OR) = 1.063, 95 % confidence interval (CI): 1.017–1.112; *P* = 0.002, OR = 1.201, 95 % CI: 1.068–1.351; *P* = 0.001, OR = 4.717, 95 % CI: 1.967–11.315) (Table 4).

**Table 3:** Univariate analysis of risk factors for the survival of ACLF patients

Related factors	Survivor (*n* = 76) (Mean ± SD)	Non-survivor (*n* = 55) (Mean ± SD)	*t* or *χ* ^2^ value	*P* value
Male (%)	64 (84.21)	47 (85.45)	0.04	0.85
HBeAg positive (%)	19 (25.00)	18 (32.73)	0.94	0.33
Treatment without NAs (%)	30 (39.47)	40 (72.73)	14.18	<0.001
Age (yr)	39.49 ± 10.50	45.05 ± 9.96	3.06	0.003
HBV DNA log_10_ (copies/ml)	5.52 ± 1.40	5.50 ± 1.60	0.08	0.94
TBIL (μmol/L)	271.33 ± 111.05	265.23 ± 122.78	0.30	0.77
Albumin (g/L)	34.67 ± 5.44	34.34 ± 4.77	0.37	0.71
ALT (U/L)	912.92 ± 861.61	950.50 ± 744.03	0.26	0.80
PTA (%)	33.81 ± 9.40	30.21 ± 10.50	2.05	0.042
Creatinine (μmol/L)	66.66 ± 14.74	79.03 ± 57.78	1.55	0.13
MELD Score	22.11 ± 2.84	25.24 ± 6.19	3.49	0.001

**Table 4:** Multivariate analysis of risk factors for the survival of ACLF patients

Related factors	Regression coefficient (B)	Standard error	Wals	*P*	OR value	95 % CI
Age (yr)	0.061	0.023	7.355	0.007	1.063	1.017-1.112
Treatment without NAs	1.551	0.446	12.077	0.001	4.717	1.967-11.315
HBV DNA log_10_ (copies/ml)	0.310	0.164	3.584	0.058	1.363	0.989-1.879
MELD score	0.183	0.060	9.356	0.002	1.201	1.068-1.351
Cons	-9.717	2.306	17.761	<0.001	-	-

## Discussion

ACLF is a serious liver disease caused by multiple factors, with impairment of several major liver functions, including synthetic function, detoxification and metabolic regulation. In addition, it can lead to ascites, jaundice, cholestasis, bleeding, hepatic encephalopathy, and hepatorenal syndrome [5]. Fatality rate of ACLF is high and the prognosis of ACLF is poor [6].

Previous research has revealed that HBV replication and high serum HBV load are key factors for severe impairment of liver function and associated with rapid disease progression of ACLF. Previously research revealed that antiviral treatment of ACLF could effectively decrease fatality rate and improve patients’ quality of life [27]. A study from Taiwan suggested that antiviral therapy increases short-term survival rate of ACLF at the early stage of liver failure, although it is not able to increases short-term survival rate of ACLF at the late stage of liver failure. Antiviral treatment as early as possible is more effective in represses viral replication, preventing deterioration of clinical condition and improving prognosis of ACLF. Lamivudine has been proven effective in inhibiting viral replication, improving biochemical and histology, and reducing the inflammatory response. Lamivudine treatment has been shown useful for the patients with HBV-related ACLF [28]. Adefovir dipivoxil, a phosphonate nucleoside analog of AMP, represses polymerase activity of HBV [23, 29], and suppresses replication of wild-type and lamivudine-resistant HBV. Previous research has suggested that combination antiviral treatment with lamivudine and adefovir dipivoxil is more effective at decreasing serum HBV load in chronic hepatitis B patients than using one nucleoside [25, 26]. ACLF usually develops rapidly, and patients are in poor condition and have a poor prognosis. Initial combination antiviral treatment with lamivudine and adefovir dipivoxil can improve prognosis of ACLF through repression HBV replication rapidly and effectively. Until recently, initial combination antiviral treatment in ACLF patients has rarely been reported. This prompted us to investigate the effect of initial combination antiviral treatment with lamivudine plus adefovir dipivoxil in ACLF patients.

In our study, the TBIL, ALB and PTA were significant improved at 1 week to 4 week in the NA group. While in the non-NA group, the TBIL, ALB and PTA were not effectively improved as the duration of treatment. Previous research showed that the 3-month accumulated fatality rate in patients treated with entecavir, lamivudine and non-NAs was 51.51 %, 50 % and 59.46 %, respectively. The difference in 3-month accumulated fatality rate between patients treated with NAs or not was not significant [7]. Another research reported that the 3-month accumulated fatality rate in patients treated with entecavir, lamivudine and non-NAs was 30.95 %, 40 % and 64.71 %, respectively [8]. In this study, patients treated with NAs had a significantly lower accumulated fatality rate than patients treated without NAs. Our research suggested that the accumulated fatality rates in the NA group were lower than in the non-NA group at weeks 2–4 and 12, and that these differences were significant. The 12-week accumulated fatality rate was 24.59 % and 57.14 % in the NA and non-NA group, respectively. In our study,univariate analysis showed that age, MELD score, PTA and treatment without NAs were risk factors for short-term survival of ACLF patients. Multivariate analysis by unconditional logistic regression suggested that age, MELD score and treatment without NAs were independent risk factors for fatality rate at short-term in ACLF patients. Our results showed that initial combination antiviral treatment significantly decreased short-term accumulated fatality rate of ACLF patients. It is likely that initial combination antiviral treatment with lamivudine plus adefovir dipivoxil may be more effective in inhibiting HBV replication, decreasing serum HBV DNA loads, lessening inflammation impairment in hepatic tissue, and improving prognosis.

Our study has potential weakness. First, our research is a retrospective research during May 2006 to June 2011. At that time, lamivudine and adefovir dipivoxil are the most widely used nucleoside analogues in China. So, our study only investigated the initial antiviral treatment with lamivudine plus adefovir dipivoxil. In the future, we should investigate antiviral treatment effect of other nucleoside analogues such as tenofovir, entecavir and telbivudine in ACLF patients. Second, the sample size of our research is relatively small. Next step, we should verify our results in a large sample size.

## Conclusions

Our results revealed that initial combination antiviral treatment with lamivudine plus adefovir dipivoxil is able to improve biochemical parameters, increase PTA and decreased short-term fatality rate of ACLF patients.

## Methods

### Patients

ACLF was diagnosed according to “Diagnostic and Treatment Guidelines for Liver Failure (2012 version)” recommended by the Chinese Medical Association [4]. The inclusion criteria were as follows: (1) age 18–65 years; (2) hepatitis B surface antigen (HBsAg) positive for >6 months; (3) serum HBV DNA level >3 log_10_ copies/ml; (4) recent development of increasing jaundice (serum bilirubin concentration >171 μmol/l) and decreasing plasma PTA (≤40 %) or INR >1.5; and (5) recent development of complications such as hepatic encephalopathy, abrupt and obvious increase in ascites, spontaneous bacterial peritonitis, or hepatorenal syndrome.

Patients affected by other viral hepatitis, alcohol hepatitis, autoimmune hepatitis, drug-induced hepatitis, primary liver cancer and hepatorenal syndrome were excluded. Patients with jaundice caused by obstructive or hemolytic diseases or prolonged prothrombin time induced by blood system diseases were also excluded.

A total of 131 eligible HBV-related ACLF patients were included in our retrospective study. Seventy patients hospitalized in the Department of Infectious Diseases, People’s Hospital of Deyang City from May 2006 to January 2009, were not treated with NAs and assigned to the non-NA group. Sixty-one patients hospitalized from February 2009 to June 2011 were treated with lamivudine plus adefovir and assigned to the NA group. No patients were previously treated with nucleoside analogues and interferon. Our study protocol conformed to the ethical guidelines of the 1975 Declaration of Helsinki and was approved by the Ethics Committee of People’s Hospital of Deyang City.

### Treatment schedule

All patients received standard medical treatment, including supply of energy, fresh plasma and albumin, maintenance of water, electrolyte and acid–base equilibrium, promoting growth of hepatic cells, anti-inflammatory and antioxidant treatments, and liver protection methods. Patients with spontaneous peritonitis received anti-infective therapy using third-generation cephalosporins. Besides standard internal medical therapy, patients in the NA group received initial combination antiviral treatment with lamivudine (100 mg daily) (GlaxoSmithKline, Greenford, UK) plus adefovir dipivoxil (10 mg daily) (GlaxoSmithKline) within 24 h after hospitalization.

### Measurement of serum markers

Serum markers reflecting hepatocyte damage, including serum ALT, albumin, TBIL, and creatinine, were assayed by colorimetry (Hitachi 7180; Hitachi, Tokyo, Japan). Serum HBV markers, including HBsAg, hepatitis B surface antibody, HBeAg, hepatitis B e antibody and hepatitis B core antibody, were detected by time-resolved fluorescence immunoassay (Tailai-II, Fenghua, Guangzhou, China). PTA was measured by solidification method and INR was calculated according to the manufacturer’s instructions (Sysmex CA 7000; Sysmex, Kobe, Japan). Serum HBV DNA load was quantified by Quantitive Diagnostic Kit for Hepatitis B Virus DNA (Qiagen, Shenzhen, Guangdong, China). The MELD score was calculated using the equation: MELD score = 3.78 × ln [total bilirubin (mg/dL)] + 11.2 × ln INR + 9.57 × ln [creatinine (mg/dL)] + 6.43 [30].

### Follow-up

Patients were followed up at weeks 1–4 and 12. Clinical and laboratory data and adverse events were recorded.

### Statistical analyses

Statistical analyses, including Student’s *t* test, *χ*^2^ test and unconditional logistic regression, were performed by SPSS version 17.0. *P* < 0.05 was considered statistically significant.

## Abbreviations

ACLF: Acute-on-chronic liver failure
ALT: Alanine aminotransferase
HBeAg: Hepatitis B e antigen
HBsAg: Hepatitis B surface antigen
HBV: Hepatitis B virus
INR: International normalized ratio
MELD: Model for end-stage liver disease
NA: Nucleoside analog
PTA: Prothrombin activity
TBIL: Total bilirubin
